# Prognostic Impact of Proteinuria at Manifestation in Adult Nephrotic Syndrome Patients: Insights from a Prospective Cohort Study

**DOI:** 10.7759/cureus.62143

**Published:** 2024-06-11

**Authors:** Srinivas Nagaram, Priscilla Charles, Hanumanthappa Nandeesha, Sreejith Parameswaran, Palanivel Chinnakali, Rajesh Nachiappa Ganesh

**Affiliations:** 1 Pathology, Jawaharlal Institute of Postgraduate Medical Education & Research, Puducherry, IND; 2 Biochemistry, Jawaharlal Institute of Postgraduate Medical Education & Research, Puducherry, IND; 3 Nephrology, Jawaharlal Institute of Postgraduate Medical Education & Research, Puducherry, IND; 4 Preventive Medicine, Jawaharlal Institute of Postgraduate Medical Education & Research, Puducherry, IND

**Keywords:** proteinuria, pyrogallol red method, chronic kidney disease, estimated glomerular filtration rate, nephrotic syndrome

## Abstract

Background and objective

Nephrotic syndrome is a significant worldwide health concern impacting millions of people and is marked by heavy proteinuria, edema, and decreased serum levels of albumin. Albuminuria arises from abnormal glomerular permeability and impaired tubular reabsorption, contributing to declining kidney function and a heightened risk of cardiovascular complications. The objective of this study was to investigate the prognostic role of proteinuria on the persistent decline in estimated glomerular filtration rate (eGFR) (<30 ml/minute/1.73m^2^) during follow-up and the dynamics of remission and relapse in various subtypes of nephrotic syndrome.

Methods

A total of 134 adult patients, diagnosed with various histopathological categories of nephrotic syndrome, were prospectively studied. Urine protein levels were assessed using the pyrogallol red-molybdate (PRM) method. The Kaplan-Meier analysis and log-rank test were utilized to assess the prognostic role of proteinuria at manifestation on persistent decline in estimated glomerular filtration rate (eGFR) (<30 ml/minute/1.73m^2^) and to evaluate remission and relapse based on proteinuria levels over an 18-month follow-up period.

Results

Patients with sub-nephrotic levels of proteinuria at manifestation did not progress to end-stage renal disease on follow-up. Patients with sub-nephrotic levels of albuminuria at manifestation were significantly associated with remission on follow-up. The Kaplan-Meier analysis indicated a significant probability of persistent eGFR decline (p < 0.001) in adult nephrotics with higher levels of albuminuria. Furthermore, patients with sub-nephrotic range proteinuria had earlier remission (p < 0.001) compared to those with relapse (p = 0.001) during the follow-up, as demonstrated by log-rank tests.

Conclusion

This study highlights that sub-nephrotic albuminuria at manifestation is linked to a reduced risk of renal progression and persistent eGFR decline compared to adult nephrotics with higher levels of albuminuria. Early detection and effective management of proteinuria, are crucial for preventing renal function decline and improving patient outcomes.

## Introduction

Nephrotic syndrome is a clinical condition characterized by > 3.5 grams of albuminuria, edema, and hypoalbuminemia [1]. Proteinuria, which is the cardinal sign of nephrotic syndrome, is considered a significant healthcare concern impacting millions of people globally [2,3]. It results from two mechanisms: firstly, the abnormal passage of albumin across the glomerular capillary basement membrane due to increased permeability, and secondly, the subsequent impairment in protein reabsorption by epithelial cells in the proximal tubules. In different glomerular diseases, the structural integrity of the glomerular capillary wall, which is typically impermeable to albumin, is compromised, allowing large molecular weight proteins to enter the tubular lumen [4].

The extent of albuminuria is a sensitive indicator of progressive renal dysfunction and is recognized as an independent risk factor for cardiovascular (CV) morbidity and mortality [3]. In clinical glomerulopathies in humans, the presence and extent of proteinuria are linked to the advancement of renal failure [5]. Moreover, it is widely acknowledged that microalbuminuria (defined as urinary albumin excretion ranging from 30 mg to 300 mg per day) acts as an initial indicator of renal involvement in conditions such as diabetes, obesity, and metabolic syndrome. However, microalbuminuria often transitions to overt proteinuria in 10% to 50% of patients and progresses to end-stage kidney disease, ultimately necessitating dialysis or transplantation [3].

Chronic kidney disease (CKD) has become a major public health concern worldwide in recent decades. The CKD prevalence is substantial, estimated between 11% and 13%, with glomerular disease (GD), particularly primary glomerular disease (PGD), ranking among the primary contributors to CKD [6]. PGDs like membranous nephropathy (MN), focal segmental glomerulosclerosis (FSGS), and IgA nephropathy (IgAN) are linked to a more significant decline in kidney function. The rate of decline is closely linked to the extent of proteinuria, the severity of arterial hypertension, and GFR during the time of diagnosis [7].

Chronic proteinuric glomerulopathies exhibit a permanent impairment in the selective protein filtration ability of the glomerular barrier, leading to progressive glomerulosclerosis [8]. The pathogenesis of nephrotic syndrome commonly involves deposition or entrapment of immune complexes affecting the glomerular capillaries and podocytes, and results in extensive proteinuria and hypoproteinemia. The exact pathophysiologic mechanisms underlying heavy proteinuria in various diseases are still under extensive research [9]. This study focused on investigating the impact of proteinuria on the persistent decline in estimated glomerular filtration rate (eGFR) (<30 ml/minute/1.73m²) and the dynamics of remission and relapse in various nephrotic syndrome subtypes. Understanding these relationships is crucial for developing targeted therapeutic strategies and improving patient outcomes.

## Materials and methods

Study design

The study aimed to assess the correlation between proteinuria levels at presentation in adult nephrotics and the predictive and prognostic significance concerning the eGFR decline, relapse, and remission of nephrotic syndrome during follow-up. We designed a longitudinal cohort study of adult nephrotic syndrome patients, including both de novo newly diagnosed patients as well as those at various stages of the disease, and followed them up consecutively monitoring the various renal function parameters and in particular the rate of and persistent decline in eGFR. The research design involved a longitudinal approach, observing changes in proteinuria levels and eGFR over time, and analyzing their impact on the progression of nephrotic syndrome. The study was conducted at the Pathology Department, Jawaharlal Institute of Medical Education and Research (JIPMER), Puducherry, India, following approval from the JIPMER Ethics Committee (approval number: JIP/IEC/2019/070). It strictly followed the ethical guidelines set in the Declaration of Helsinki.

Characteristics of study participants

We enrolled a total of 134 patients aged 18 years and above, diagnosed with various categories of biopsy-proven nephrotic syndrome, after obtaining informed consent from the patients in the Nephrology Department. The distribution included membranous glomerulonephritis (MGN, n = 50), minimal change disease (MCD, n = 28), FSGS (n = 25), IgAN (n = 11), infection-related glomerulonephritis (IRGN, n = 5), systemic lupus nephritis (SLEN, n = 5), C3 glomerulopathy (C3G, n = 4), pauci-immune crescentic glomerulonephritis or anti-neutrophil cytoplasmic antibodies (ANCA)-associated crescentic glomerulonephritis (CGN) (ANCA CGN, n = 3), immune complex-mediated glomerulonephritis (ICGN, n = 2), and light chain amyloidosis (LCA, n = 1).

Demographic characteristics such as age and gender, along with biochemical parameters at diagnosis, were meticulously documented for each patient. The outcome measure of persistent decline in eGFR was assessed utilizing the CKD-Epidemiology Collaboration (CKD-EPI) creatinine 2009 equation and the National Kidney Foundation application software. Definitions for remission and relapse were based on the 2021 KDIGO (Kidney Disease: Improving Global Outcomes) Clinical Practice Guideline for Managing Glomerular Diseases. Complete remission (CR) is defined as having proteinuria < 0.3 g/day, normal serum albumin levels (≥ 3.5 g/dl), and stable kidney function. Relapse is characterized by proteinuria exceeding 3.5 g/day after complete remission or by a proteinuria increase of more than 50% during partial remission [10].

Measurement of urinary protein

We employed the pyrogallol red-molybdate (PRM) method manufactured by Beckman Coulter, Inc., Brea, California, United States, original reference code: OSR6170, for the quantitative determination of urine protein analyzed by the AU680 chemical analyzer (Beckman Coulter, Inc., Brea, California, United States). Pyrogallol red, when combined with molybdate, forms a red complex that has a maximum absorbance at 470 nm. This assay leverages the shift in absorbance that occurs when the pyrogallol red-molybdate complex binds to the basic amino groups of protein molecules. In the presence of protein, this binding results in the formation of a blue-purple complex with a maximum absorbance at 600 nm. The absorbance of this blue-purple complex is directly proportional to the protein concentration in the sample. This method is linear from 4 to 200 mg/dL. Samples that exceeded the upper limit of linearity were diluted with water and retested.

Statistical analysis

The distribution of the data was assessed using the Shapiro-Wilk test. Continuous variables were summarized using either mean ± standard deviation (SD) or median with interquartile range (IQR, 25th and 75th percentile). The Mann-Whitney U test was employed to evaluate the association between proteinuria levels among patients in remission and those experiencing relapse. Time-to-event analysis was performed using the Kaplan-Meier analysis with a log-rank test, focusing on endpoints such as a persistent decline in eGFR, remission, and relapse based on proteinuria levels (sub-nephrotic, overt, and nephrotic ranges). All analyses were conducted using IBM SPSS Statistics for Windows, Version 19, (Released 2010; IBM Corp., Armonk, New York, United States) and GraphPad Prism version 8.0.2 (Insight Partners, New York City, New York, United States). A p-value of less than 0.050 was considered statistically significant.

## Results

The baseline demographic and biochemical characteristics of the study participants at enrollment are presented according to the stages of CKD (Table 1).

**Table 1:** Demographic and biochemical parameters according to eGFR levels (CKD stages) MAP: mean arterial pressure; UPCR: urine protein-to-creatinine ratio; AST: aspartate aminotransferase; ALT: alanine aminotransferase; ALP: alkaline phosphatase; WBC: White blood cell count; CKD: chronic kidney disease; eGFR: estimated glomerular filtration rate

Parameter	CKD Stage 1	CKD Stage 2	CKD Stage 3	CKD Stage 4	CKD Stage 5
Age	32.94 ± 10.98	39.71 ± 12.88	42.85 ± 13.05	36.56 ± 17.11	35.67 ± 21.07
MAP (mm Hg)	93.17 ± 11.09	90.95 ± 8.47	96.61 ± 15.86	90.50 ± 15.69	102.22 ± 13.80
UPCR (mg/mg)	1.76 (0.28 - 6.35)	4.05 (1.16 - 8.87)	3.60 (1.60 - 6.82)	4.50 (2.25 - 9.75)	4.56 (3.11 - 9.78)
Serum albumin (gm/dL)	3.08 ± 1.07	3.18 ± 0.75	2.57± 1.21	2.25 ± 1.03	2.90 ± 1.55
Total protein (gm/dl)	6.09 ± 1.10	5.47 ± 1.34	5.69 ± 1.39	5.60 ± 1.08	5.60 ± 0.56
Blood glucose (mg/dL)	93 (87 - 106)	92 (80 - 106)	106 (92 - 130)	109 (87 - 118)	114 (102 - 120)
Blood urea (mg/dL)	22 (19 - 28)	35 (28 - 44)	43 (33 - 76)	84 (55 - 96)	96 (54 - 115)
Serum creatinine	0.91 (0.63 - 1.00)	1.09 (1.00 - 1.33)	1.79 (1.38 -2.18)	3.71 (2.16 - 4.36)	3.14 (2.5 - 5.21)
Sodium (mEq/L)	137.65 ± 2.54	136.80 ± 2.14	136.70 ± 3.41	136.38 ± 2.17	136.45 ± 3.12
Potassium (mEq/L)	4.07 ± 0.32	4.29 ± 0.29	4.30 ± 0.55	4.59 ± 0.83	4.27 ± 0.20
Calcium (mg/dL)	8.97 ± 2.17	8.63 ± 0.83	8.72 ± 0.95	8.21 ± 0.69	8.07 ± 1.24
Magnesium (mg/dL)	1.89 ± 0.25	2.01 ± 0.18	2.03 ± 0.20	2.17 ± 0.42	2.26 ± 0.28
Phosphorus (mg/dL)	3.99 ± 0.58	4.01 ± 0.92	4.15 ± 1.21	4.67 ± 0.93	4.96 ± 0.98
Chloride (mEq/L)	102.04 ± 7.56	102.84 ± 6.52	102.88 ± 9.13	104.15 ± 4.61	100.11 ± 8.26
Uric acid (mg/dL)	5.74 ± 1.75	6.45 ± 1.08	7.43 ± 1.75	7.84 ± 2.07	7.51 ± 0.71
Total bilirubin (mg/dL)	0.46 (0.33 - 0.65)	0.44 (0.31 - 0.61)	0.42 (0.28 - 0.57)	0.39 (0.29 - 0.51)	0.52 (0.39 - 0.68)
Direct bilirubin (mg/dL)	0.09 (0.07 - 0.13)	0.55 (0.05 - 0.10)	0.08 (0.06 - 0.15)	0.07 (0.05 - 0.13)	0.07 (0.05 - 0.015)
AST (IU/L)	21.07 (18.12 - 26.33)	23.25 (19.10 - 26.25)	23.25 (17.51 - 27.75)	20.66 (17.50 - 24.26)	35.53 (18.76 - 42.41)
ALT (IU/L)	19.50 (15.18 - 25.33)	17.10 (12.87 - 21.25)	17.58 (13.25 - 22.50)	16.55 (12.50 - 20.50)	37.22 (18.21 - 43.25)
ALP (IU/L)	83.75 (70.51 - 105.22)	80.60 (66.75 - 99.08)	102.12 (76.50 - 151.37)	72.13 (64.50 - 107.55)	135.37 (65.51 - 140.33)
Total cholesterol (mg/dL)	284.50 (185 - 294)	326 (192 - 379)	275 (172 -354)	301 (182 - 406)	279 (196 - 339)
Hemoglobin (gm/dL)	12.33 ± 2.25	10.75 ± 1.87	10.70 ± 2.16	10.31 ± 1.68	9.06 ± 2.40
WBC count (cells/μL)	11.08 ± 6.40	10.54 (7.75 - 13.50)	9.11 (7.33 - 11.15)	9.30 (6.95 - 10.21)	9.35 (6.52 - 11.75)

Several biochemical parameters were categorized for patients in CKD stage 1 versus stage 4. We observed that as compared to those in stage 1 CKD, patients in stage 4 CKD exhibited decreased serum albumin (3.08 ± 1.07 vs. 2.25 ± 1.03g/dL), total protein (6.09 ± 1.10 vs. 5.60 ± 1.08 g/dL) and hemoglobin (12.33 ± 2.25 vs. 10.31 ± 1.68) alongside increased urinary protein to creatinine ratio (UPCR) (1.76 (0.28 - 6.35) vs. 4.50 (2.25 - 9.75) mg/mg), blood urea (22 (19 - 28) vs. 84 (55 - 96) mg/dL), serum creatinine (0.91 (0.63 - 1.00) vs. 3.71 (2.16 - 4.36) mg/dL), and total cholesterol (284.50 (185 - 294) mg/dL) vs. 301 (182 - 406) mg/dL), with a persistent decline in eGFR. 

**Figure 1:**
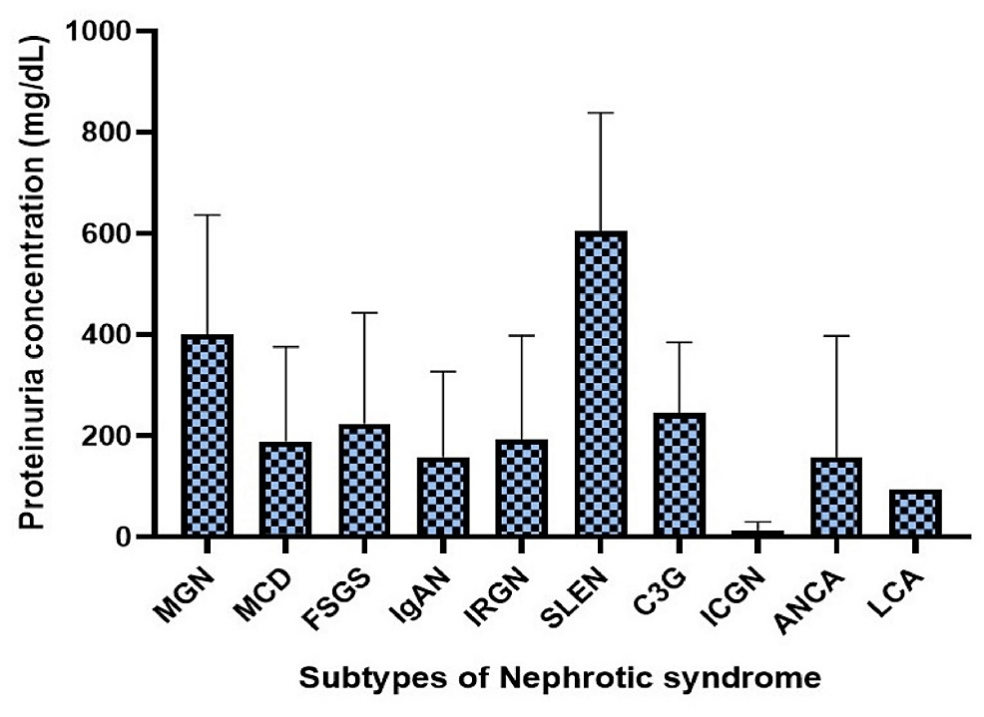
Proteinuria levels at manifestation by pathological subtypes of nephrotic syndrome MGN: membranous glomerulonephritis MCD: minimal change disease; FSGS: focal segmental glomerulosclerosis; IgAN: IgA nephropathy; IRGN: infection-related glomerulonephritis; SLEN: systemic lupus erythematosus nephritis; C3G: C3 glomerulopathy; ICGN: immune complex glomerulonephritis; ANCA: anti-neutrophil cytoplasmic antibodies; LCA: light chain amyloidosis

The urine protein levels at the time of manifestation for different pathological subtypes of nephrotic syndrome are given in Figure 1. This figure provides a detailed comparison of the initial proteinuria levels across various subtypes, highlighting the differences in urine protein levels among patients diagnosed with these distinct histopathological categories of nephrotic syndrome.

**Figure 2:**
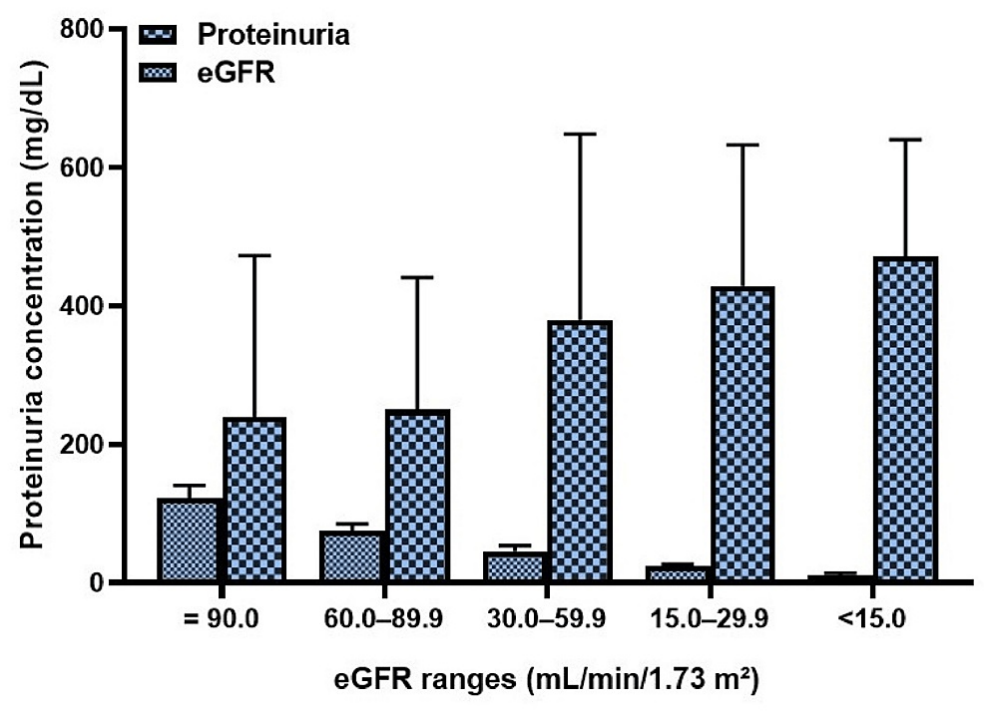
Proteinuria levels at manifestation categorized by proportionate decrease in eGFR (mL/minute/1.73m2) eGFR: estimated glomerular filtration rate

The mean eGFRs at enrollment in subtypes of nephrotic syndrome are as follows: MGN (85 ± 40), MCD (114 ± 35), FSGS (113 ± 27), IgAN (83 ± 42), IRGN (82 ± 20), SLEN (87 ± 62), and C3G (64 ± 47). Higher proteinuria levels at manifestation correspond to a faster decrease in eGFR (Figure 2).

**Figure 3:**
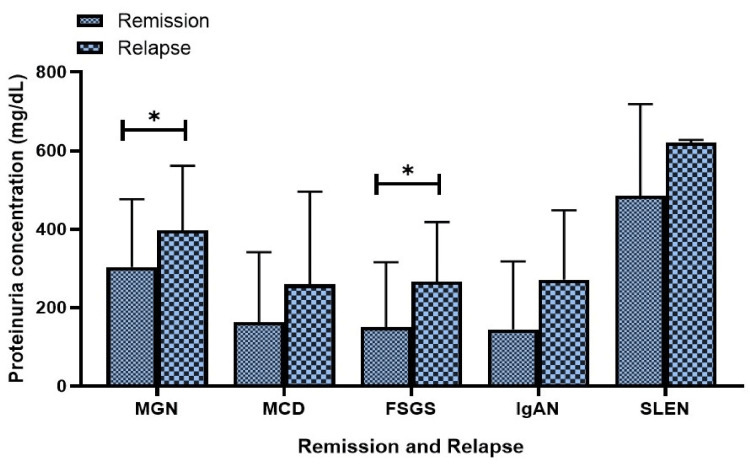
Proteinuria levels at manifestation in patients who achieved remission and relapse on follow-up MGN: membranous glomerulonephritis MCD: minimal change disease; FSGS: focal segmental glomerulosclerosis; IgAN: IgA nephropathy; SLEN: systemic lupus erythematosus nephritis

Sub-nephrotic levels of proteinuria were observed in patients experiencing remission in conditions such as MGN, MCD, FSGS, IgAN, and SLEN. Statistical significance was noted in the follow-up analysis of proteinuria levels at manifestation, among patients with remission and relapse specifically in MGN (p = 0.034) and FSGS (p = 0.028) (Figure 3).

**Figure 4:**
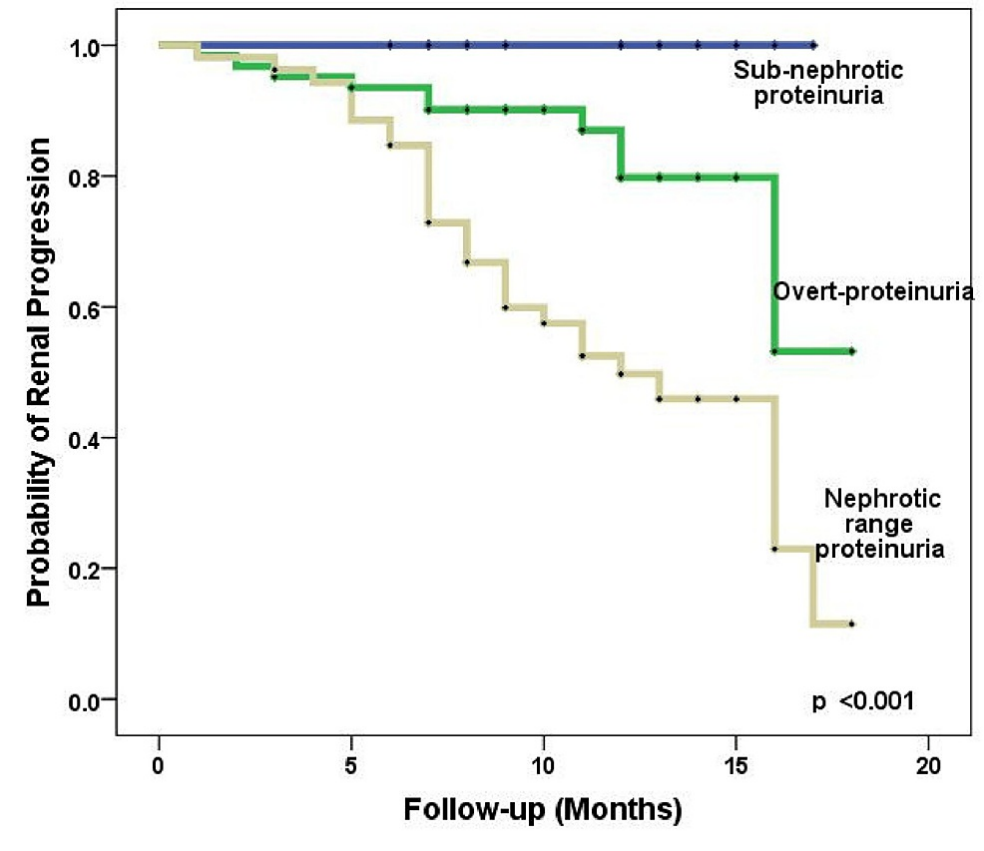
Kaplan-Meier analysis of the impact of proteinuria at manifestation on the probability of persistent eGFR decline eGFR: estimated glomerular filtration rate

There was no renal progression or a persistent decline in eGFR observed in patients with sub-nephrotic range proteinuria when compared to those with nephrotic range proteinuria. A statistically significant difference was found in the log-rank test during the Kaplan-Meier analysis for the probability of a persistent decline in eGFR (p < 0.001) (Figure 4).

**Figure 5:**
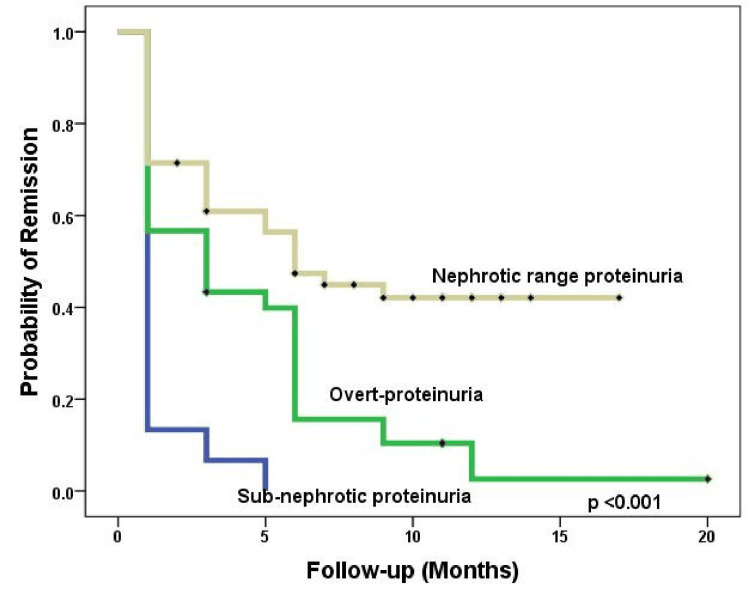
Kaplan-Meier analysis of the impact of proteinuria at manifestation on the probability of remission

**Figure 6:**
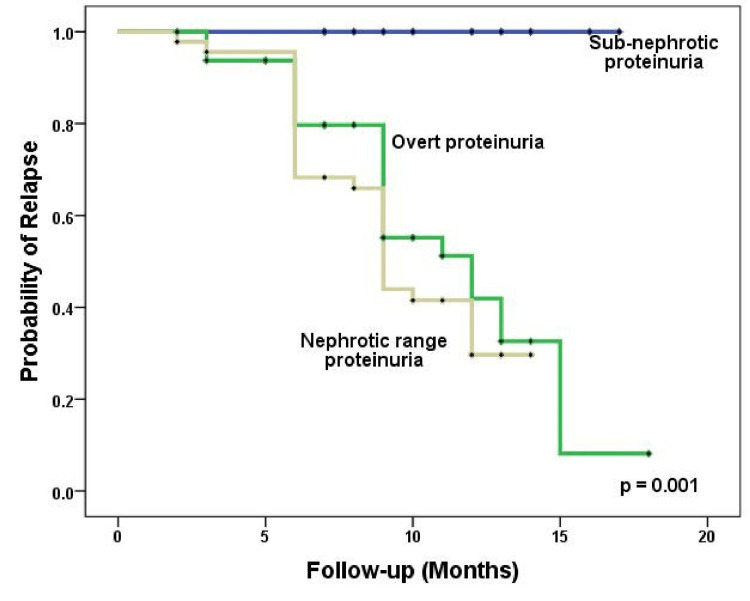
Kaplan-Meier analysis of the impact of proteinuria at manifestation on the probability of relapse

Additionally, patients with sub-nephrotic range proteinuria experienced early remission (p < 0.001) (Figure 5) compared to relapse (p = 0.001) (Figure 6), as indicated by log-rank tests.

## Discussion

In this longitudinal observational cohort study, no renal progression or persistent decline in eGFR was observed in patients with sub-nephrotic range proteinuria compared to those with overt proteinuria, and nephrotic range proteinuria. Additionally, we observed a decrease in albumin, total proteins, and hemoglobin levels alongside an increase in UPCR, blood urea, serum creatinine, and cholesterol levels in our study subjects.

These study results align with findings from a previous investigation involving 927 patients, which indicated that kidney function in individuals with normal-range proteinuria carried a significantly lower risk of progression than those with abnormal-range proteinuria [11]. The inverse correlation of serum albumin, hemoglobin, and total proteins with UPCR aligns with a study by Li et al., which demonstrated similar results in patients with nephrotic syndrome [12]. Multiple studies have found a link between proteinuria and renal function decline. The degree of proteinuria, particularly in glomerular diseases, is closely associated with the advancement of CKD and the rate of decline of albuminuria, with treatment linked to a less rapid decrease in the eGFR, leading to an improved prognosis for renal function [7].

Ruggenenti et al. conducted a study of 352 patients with non-diabetic kidney disease and found that those with proteinuria levels exceeding 3.9 g/24 hours at diagnosis experienced a more significant decline in GFR compared to patients with proteinuria levels below 1.9 g/24 hours. Additionally, these individuals had a higher likelihood of progressing to end-stage kidney disease (ESKD) [13]. A study of 2420 diabetic patients found that those diagnosed with microalbuminuria had a 2.1-fold increased risk of developing ESKD. In contrast, individuals with overt proteinuria were 9.1 times more likely to progress to ESKD than those without proteinuria [14]. A mass screening of 107192 participants over the age of 18 in Okinawa, Japan, revealed that proteinuria was the strongest predictor of ESRD risk over a ten-year period among the general population [15].

MGN is the primary type of adult nephrotic syndrome and is less common in children. About 30% of MGN cases progress to ESRD. MGN involves autoantibodies and complement activation, forming an immune complex targeting and damaging the glomerular filtration barrier, and the resultant proteinuria. MCD and FSGS are conditions associated with podocyte injury, manifesting with significant proteinuria [6]. We found that patients experiencing a relapse in MGN and FSGS had considerably greater levels of proteinuria at manifestation. Lower serum albumin and eGFR were linked to delayed remission in MCD patients. However, they were not associated with relapse, emphasizing their role in treatment response monitoring [16]. A study found that primary MGN patients with sub-nephrotic proteinuria had a better prognosis, with about 80% achieving complete remission and only 20% redeveloping nephrotic range proteinuria during follow-up [17]. IgAN is the most frequent cause of glomerulonephritis. Studies have shown that patients with IgAN who have proteinuria levels exceeding 0.5 g/day face an increased risk of kidney failure. This risk is particularly pronounced in patients with proteinuria levels of 1.0 g/day or more before starting treatment [18]. In type 2 diabetes mellitus (T2DM), both albuminuria and eGFR are crucial predictors of ESRD and mortality. Additionally, the combined assessment of albuminuria and eGFR provides a significantly improved predictive value compared to either measure alone [14]. Apart from its connection to kidney disease advancement, proteinuria heightens the cardiovascular risk and mortality in patients with and without diabetes [19].

The measurement of proteins in human urine is a crucial diagnostic tool for renal diseases. In this study, we quantified urine protein using the PRM method. Numerous methods exist for determining urinary protein, based on colorimetric, turbidimetric, electrophoretic, or immunological principles. Turbidimetric methods include sulfosalicylic acid (SSA), sulfosalicylic acid with sodium sulfate (SSSS), trichloroacetic acid (TCA), and benzethonium chloride (BEC) methods [20]. Among the dye-binding methods, the PRM method is commonly used in most hospitals due to its higher sensitivity, precision, and practicality [20]. While methods that determine total urinary protein through precipitation and indirect biuret reactions are accurate, they are also time-consuming and therefore not widely utilized. Protein dye-binding assays, on the other hand, are rapid, simple, and easily automated [21].

This study found no renal progression or persistent eGFR decline in patients with sub-nephrotic proteinuria compared to those with overt or nephrotic-range proteinuria. Significant correlations between proteinuria and changes in serum markers were noted. However, the small sample size limits the study, and further research with larger cohorts and longer follow-ups is needed for broader validation.

## Conclusions

This longitudinal observational cohort study highlights the significant impact of proteinuria levels on renal outcomes in adult nephrotic syndrome patients. Our findings suggests that sub-nephrotic range proteinuria at initial manifestation exhibits a lower risk of renal progression to higher CKD stages and persistent decline in eGFR compared with overt proteinuria or nephrotic-range proteinuria. The results emphasize the importance of early detection and effective management of proteinuria to prevent renal function decline. Future research should focus on understanding the underlying mechanisms and developing targeted therapeutic strategies to manage proteinuria and inflammation in patients with glomerular diseases, ultimately enhancing the quality of life of the patient and prognosis of the disease condition.
